# Determination of Traffic Lane in Tunnel and Positioning of Autonomous Vehicles Using Chromaticity of LED Lights

**DOI:** 10.3390/s22082912

**Published:** 2022-04-11

**Authors:** Joo Woo, So-Hyeon Jo, Gi-Sig Byun, Sun-Young Kim, Seok-Geun Jee, Ju-Hyeon Seong, Yeon-Man Jeong, Jae-Hoon Jeong

**Affiliations:** 1 The Department of Control and Instrumentation Engineering, Pukyong National University, Busan 48513, Korea; whj9419@naver.com (J.W.); shjo960@naver.com (S.-H.J.); 2 The School of Mechanical System Engineering, Kunsan National University, Gunsan 54150, Korea; 3 The School of IT, Information and Control Engineering, Kunsan National University, Gunsan 54150, Korea; 4 The Department of Liberal Education & Interdisciplinary Major of Maritime AI Convergence, Korea Maritime & Ocean University, Busan 49112, Korea; 5 The Department of Information and Telecommunication Engineering, Gangneung-Wonju National University, Wonju 26403, Korea

**Keywords:** GPS-shaded areas, vehicle-navigation system, chromaticity, vehicle-positioning system

## Abstract

Currently, the location recognition and positioning system are the essential parts of unmanned vehicles. Among them, location estimation under GPS-denied environments is currently being studied using IMU, Wi-Fi, and VLC, but there are problems such as cumulative errors, hardware complexity, and precision positioning. To address this problem with the current positioning system, the present study proposed a lane positioning technique by analyzing the chromaticity coordinates, judging from the color temperature of LED lights in tunnels. The tunnel environment was built using LEDs with three color temperatures, and to solve nonlinear problems such as lane positioning from chromaticity analysis, a single input single output fuzzy algorithm was developed to estimate the position of an object on lanes using chromaticity values of signals measured by RGB sensors. The RGB value measured by the sensor removes the disturbance through the pre-processing filter, accepts only the tunnel LED information, and estimates where it is located on the x-distance indicating the lane position through a fuzzy algorithm. Finally, the performance of the fuzzy algorithm was evaluated through experiments, and the accuracy was shown with an average error of less than 4.86%.

## 1. Introduction

With the advancement of electric vehicles, autonomous vehicles are being studied in several papers for the convenience and safety of users. Cameras, communication, sensors, and controllers are combined in an existing car, and it is reflected in vehicle operation through recognition of the vehicle’s surroundings and the driver’s condition. As the driver’s intervention is reduced, the vehicle’s positioning system has become an essential element of autonomous driving [1,2,3,4,5,6,7,8]. Currently, the real-time positioning system for vehicles is highly dependent on GPS. However, since the signal strength is low, the efficiency is lowered in environments where GPS is rejected, such as blockages by buildings or structures and non-signal areas. Research using IMU, Wi-Fi, VLC, camera, for example, is in progress to supplement the accuracy problem of GPS [9,10,11]. Nevertheless, in the case of IMU, there is a problem with error generation according to time. When estimating the location using Wi-Fi, triangulation is used, but accuracy problems may occur due to magnetic field or sensor deviation, and an additional terminal is required. Visible light communication (VLC) attracts attention as an indoor positioning system. Compared to the problems of system instability and response time of other positioning methods using communication, VLC is stable against electromagnetic interference, so that it can be a good alternative [12]. There are various measurement methods and a unique ID is given to each LED. When using proximity, it is simple but has low accuracy, and when using methods such as signal strength or fingerprinting, the accuracy is high but complicated [12,13,14]. To improve the accuracy of the real-time positioning system for vehicles, the present study attempted to analyze the chromaticity of LED light and proposed a lane positioning technology for a tunnel. LED has the advantage of being small in size, having a long lifespan, and low power consumption compared to existing lights, so it is thought to replace many other lights. By estimating the chromaticity value according to the position, it is possible to estimate the position intuitively. It is expected that location estimation will be possible using the existing tunnel lighting infrastructure without the complex formulas and hardware infrastructure required for existing triangulation and Wi-Fi communication techniques.

In the current study, the positions of vehicles on lanes running inside a tunnel have been determined based only on the chromaticity of LED lights installed under the ceiling inside a simulator that imitates a tunnel environment. To that end, a fuzzy algorithm for analyzing the signals measured by RGB sensors was developed, and its performance was evaluated based on the experiment results. Fuzzy logic can solve nonlinear problems such as lane positioning from chromaticity analysis. The fuzzy algorithm showed excellent distinction ability in lane positioning using the output signals of an RGB sensor [1,2,3,4]. In this paper, Section 2, “Chromaticity of light” explains the Chromaticity with tristimulus values XYZ. Section 3, “System Principle” introduces “Construction of tunnel simulator” and “RGB sensor signal processing method”. Section 3.1 describes the measurement environment, and Section 3.2 explains the methods to remove disturbance for the RGB sensor. Section 4, “Experiment and Analysis” introduces “Fuzzy logic system design” and “Experimental results.” Section 4.1 describes the design of a Fuzzy system based on the chromaticity values collected. In Section 4.2, the result of the experiment is mentioned. Finally, Section 5, “Conclusions," discusses the analysis of the results and conclusion about the positioning system with color temperatures.

## 2. Chromaticity of Light

The wavelengths of the red (R), green (G), and blue (B) colors of the original stimulus are λ_R = 700.0 nm, λ_R = 546.1 nm, λ_R = 435.8 nm [1,2,3,15], respectively. The XYZ colorimetry for tristimulus values of objects is a three-color colorimetry defined by the International Commission on Illumination (CIE) for sensitivity values that are equivalent to the human eye. In the present study, the table color system with tristimulus values XYZ can be obtained through (1) using RGB values read through RGB sensors, which play the same function as the cone cells in the human eye [1,2,3,4,16].

(1) $$\left[\begin{array}{c} X\\ Y\\ Z \end{array}\right]=\left[\begin{array}{ccc} 2.7689 & 1.7517 & 1.1302\\ 1.0000 & 4.5907 & 0.0601\\ 0.0000 & 0.0565 & 5.5943 \end{array}\right]\left[\begin{array}{c} R\\ G\\ B \end{array}\right]$$

To geometrically express the vector components of the tristimulus values XYZ, a three-dimensional color space is required, but it is inconvenient to express it. Therefore, in the XYZ color space, the intersection x, y between a unit plane X + Y + Z = 1 and the color vector (X, Y, Z) can be expressed as a two-dimensional plane using the following Equation (2) [1,2,3,15].

(2) $$x=\frac{X}{X+Y+Z}\;,\;y=\frac{Y}{X+Y+Z}$$

**Figure 1:**
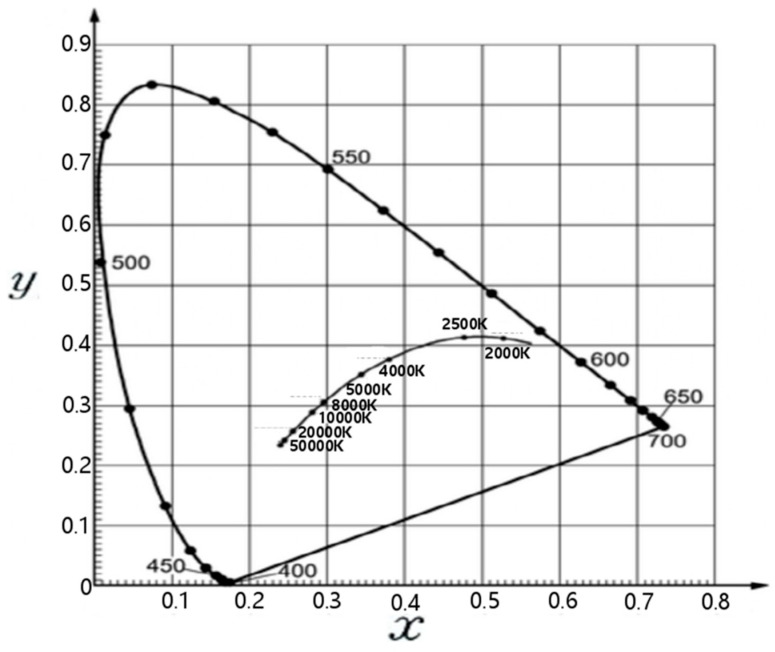
2-dimensional chromaticity diagram.

Once the chromaticity coordinate *x*, *y* of the chromaticity diagram, which is a two-dimensional plane, is determined, it can be used conveniently. Furthermore, a spectrum locus was drawn by connecting the chromaticity coordinates of the monochromatic lights, as shown in Figure 1.

**Figure 2:**
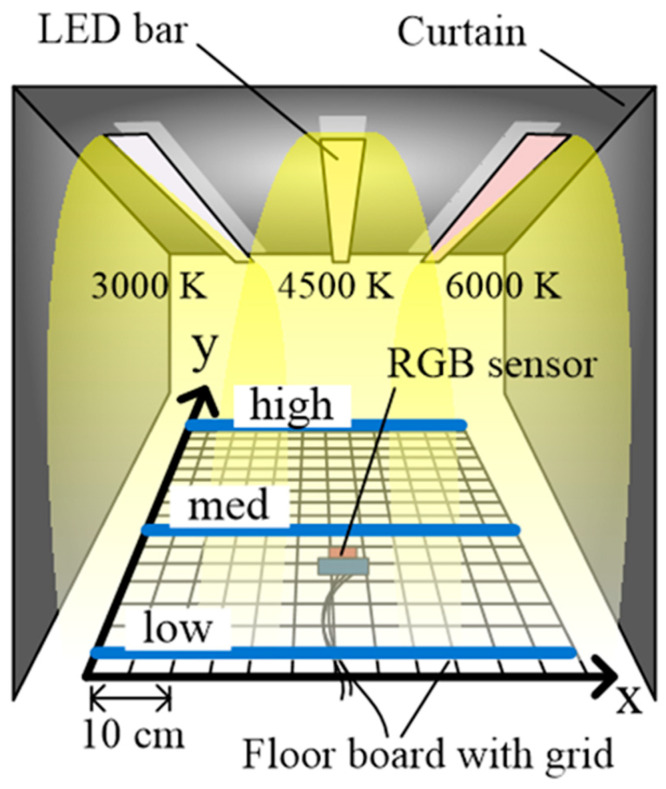
Experimental environment (tunnel simulator).

**Figure 3:**
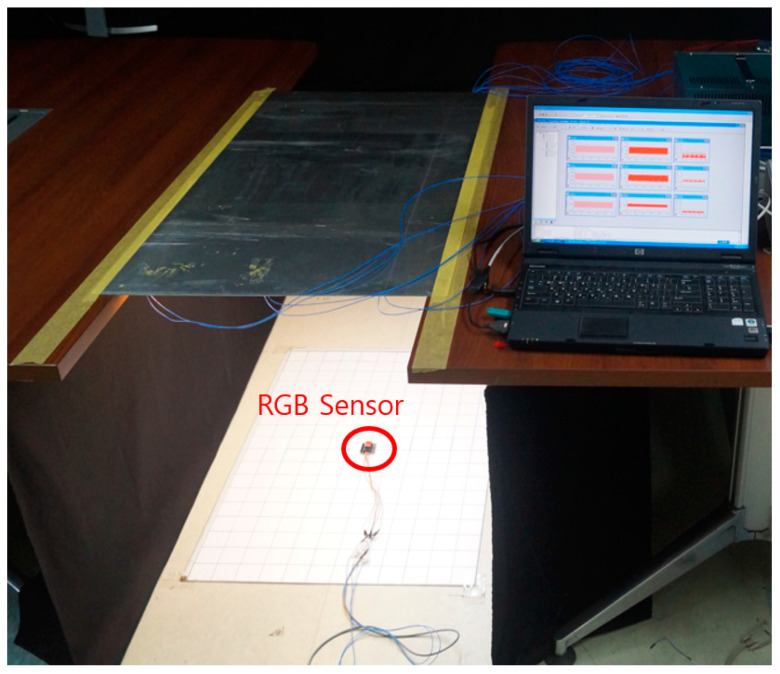
Tunnel Test-bed environment.

A blackbody is an object that emits different colors of light corresponding to its temperature. A line that connects the chromaticity points of blackbody radiation is a blackbody locus, the small locus inside the large spectrum locus, shown in Figure 1. The blackbody locus shows x and y coordinates corresponding to the color temperature (K). The black dots on the outer trajectory mean the nm wavelength of light. In this study, the LED is assumed as a blackbody. To take advantage of the color temperature characteristics of light, warm, warm white, and white LEDs were used to have different color temperatures. Three different LEDs with color temperatures of 3000 K, 4500 K, and 6000 K are used to be measured different chromaticity values corresponding to the position of the sensor, as shown in Figure 2 and Figure 3 [1,2,3,4]. In this study, x-chromaticity was used to estimate the x-distance. The reason is that among the changes in x and y between 3000 K and 6000 K in Figure 1. the 2-dimensional chromaticity diagram, x has a wider range of change than y. Therefore, it can be estimated more sensitively to x-distance changes [1,2,3,4].

## 3. System Principle

### 3.1. Construction of Tunnel Simulator

In this study, a tunnel environment simulator was built to validate the proposed positioning technique (see Figure 2). The simulator contained LED bars with the chromaticity of 3000 K, 4500 K, and 6000 K to illuminate a floorboard containing an x-axis (60 cm) and y-axis (80 cm). The interval between the LED bars was approximately 20 cm, and they were suspended at the height of 73 cm above the floorboard. The size of the square cells marked by grids on the floorboard was 5 cm × 5 cm and the chromaticity values were measured in each cell. After the LED lights were switched on, different chromaticity values were measured at each measurement site using the mixed lights of 3000 K, 4500 K, and 6000 K LEDs [1,2,3,4].

**Figure 4:**
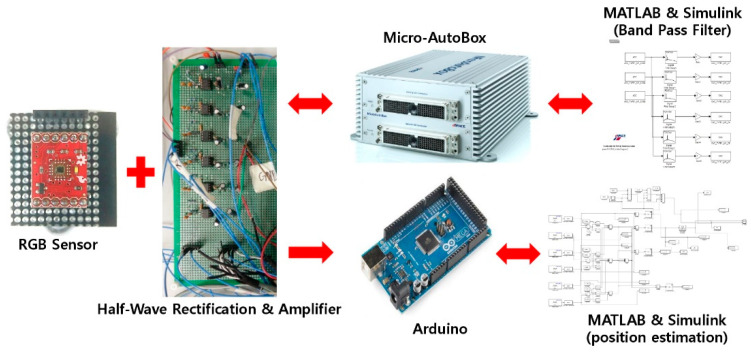
Positioning system configuration plot.

As shown in Figure 4, the entire system configuration sends the RGB signal input to the RGB sensor to Half-Wave Rectification & Amplifier. It proceeds with anti-distortion and amplification due to different light. In the Micro-AutoBox, a bandpass filter for removing different light signals for positioning is passed to derive chromaticity values and lateral position values through Arduino 2560&Matlab [1,2,3,4].

### 3.2. RGB Sensor Signal Processing Method

The RGB sensor (HDJD-s822) used in the experiment output analog voltage values. Therefore, it also reacted to other lights from the surrounding traffic roads, in addition to the LED lights for lane positioning. To solve this problem, the RGB stimulus values of the RGB sensor from LED lights for lane positioning were separated from the other lights present in the experimental environment. The LED lights for lane positioning and the other lights were distinguished by accelerating the change over time of the former in contrast to that of the latter, which changed very slowly over time [1,2,3,4]. 

**Figure 5:**
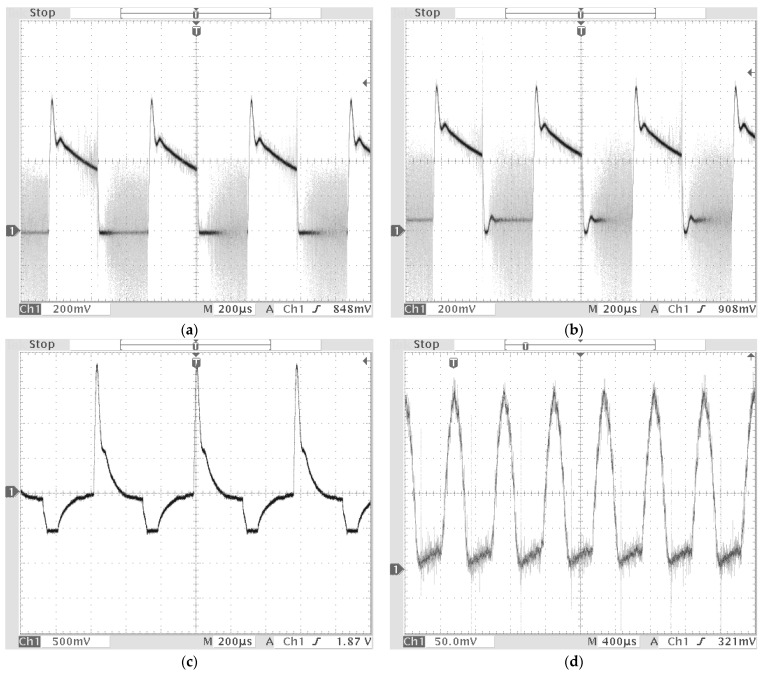
Measured RGB sensor signals during signal processing [1]. (**a**) the output signal of the RGB sensor without external disturbances. (**b**) the output signal of the RGB sensor with external disturbances. (**c**) the output signal is measured after pre-processing circuit. (**d**) the output signal is measured after the bandpass filter.

To apply this change of light over time, pulse-width modulation (PWM) of 1.75 kHz and 50% duty was applied by the LED switching driver. Figure 5 shows measured RGB sensor signals during signal processing. Figure 5a shows an output signal of the RGB sensor without external disturbances. It is different from those of external lights, such as those of car headlights and various road signboards. When the external light was irradiated together with the LED light for lane positioning, it showed distortion in the waveform by the frequency component of the disturbance and the floating component by the DC component of the disturbance. Figure 5b shows an output waveform of the RGB sensor when external disturbances are given. The floating components by the DC component of general light can be removed by using a capacitor. From the test, the floating component was removed by using a ceramic condenser (capacity: 0.0005 μF). The RGB stimulus values input to the RGB sensor passed through the preprocess circuit to remove the floating components and negative half of the signals before they are transferred to the Micro-AutoBox (ds1401), as shown in Figure 5c. To remove the frequency components of disturbances, digital filters are applied. An infinite impulse response (IIR) bandpass filter, which has fewer delay factors, is used. The bandpass filter is designed by using the MATLAB FDA tool and Simulink to pass the 1.75 kHz component. The Simulink model of this filter is interconnected with the Micro-AutoBox, and the performance of the filter is monitored in real-time. Figure 5d shows an output signal measured after filtering. Next, the output signal of the filter was converted to DC signal by using capacitors and inputs of the Arduino2560 (MCU). The calculation of x-chromaticity value and lane position using the fuzzy algorithm is also conducted by the Simulink model connected to Arduino2560 through the Embedded coder toolbox in MATLAB [1,2,3,4].

## 4. Experiment and Analysis

### 4.1. Fuzzy Logic System Design

The reference chromaticity data were required to be collected before the fuzzy controller was designed. For chromaticity measurement, the chromaticity values on three lines, low, mid, and high in Figure 2, were measured corresponding to the same x-distance value on the floor coordinates. The x-distance value was increased at 10 cm intervals until it reached 60 cm and the corresponding three chromaticity values on the three lines were continuously measured. The mean value of the three chromaticity data corresponding to each x distance value was calculated and listed in Table 1. The lane positioning was conducted corresponding to the chromaticity value by using a fuzzy system designed based on the chromaticity values in Table 1.

**Figure 6:**
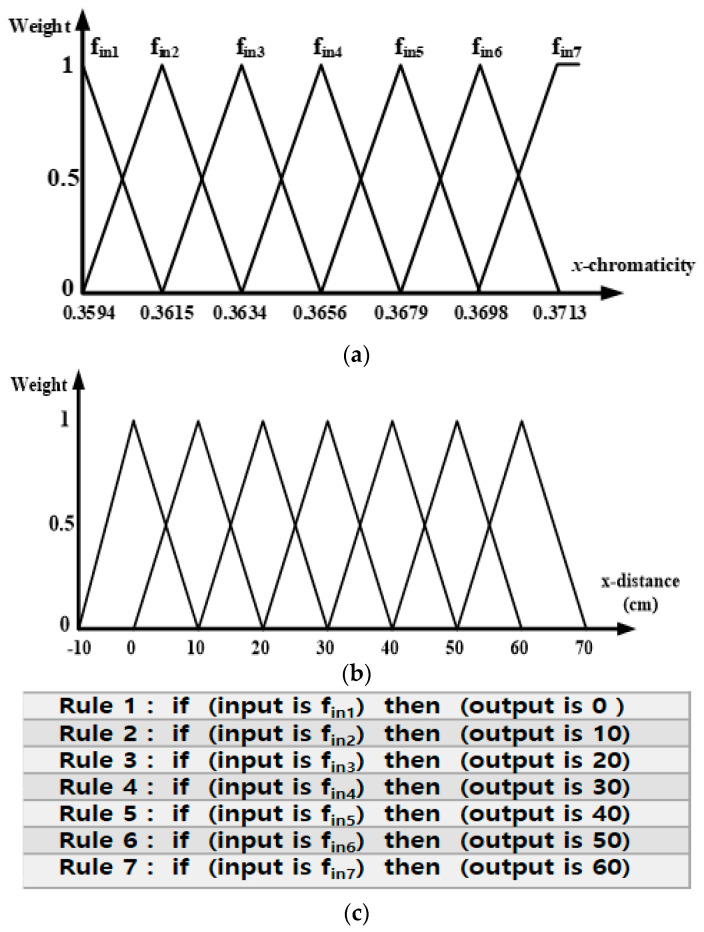
Fuzzy System. (**a**) Input membership function. (**b**) Output membership function. (**c**) 7 Fuzzy rules.

For the input of the designed single input single output (SIOS) Fuzzy system, the x-chromaticity value was defined as the input variable and the lane position, the x-distance (cm), was defined as the output. The fuzzy system, consisting of fuzzification, rule evaluation, and defuzzification procedures, was designed based on the experimental data in Table 1. Figure 6a shows the input membership function of the x-chromaticity value, which is an input variable. A triangular membership function was used in this fuzzy system. In the fuzzification procedure, x-chromaticity values were classified using the input membership function. During the procedure, the weights of the x-chromaticity values were between 0 and 1 in the triangular functions from fin1 to fin7. The rule evaluation procedure consisted of ‘if ~ then’ rule statements. This study used seven fuzzy rules (Figure 6c), which give output position values from the fuzzification procedure. To note, the rules which only have non-zero weights were activated. In the defuzzification process, the lane position was calculated from the outputs generated during the rule evaluation process. Furthermore, the weighted average technique defined by Equation (3) was used where ‘*i*’ is the number of activated rules, ‘*M*’ is the weight generated in the fuzzification procedure, and ‘*W*’ is the center values (0, 10, 20, …, 60) of triangle functions, in the output membership functions in Figure 6b, which was selected in the rule evaluation procedure [3].

(3) $$y=\frac{\sum_{i=1}^{\mathrm{Number}\;\mathrm{of}\;\mathrm{activated}\;\mathrm{rules}}\left(M^{i}\times W^{i}\right)}{\sum_{i=1}^{\mathrm{Number}\;\mathrm{of}\;\mathrm{activated}\;\mathrm{rules}}W^{i}}$$

### 4.2. Experimental Results

The designed fuzzy system was added to the Simulink model for lane positioning and interconnected with Arduino2560 (MCU).

**Figure 7:**
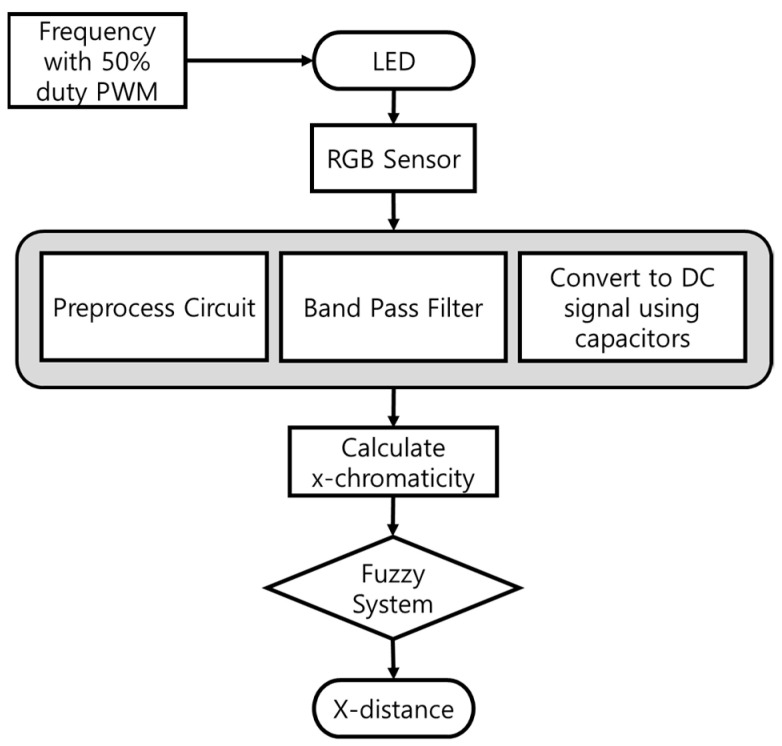
System diagram of Positioning of Autonomous Vehicles.

Figure 7 shows the overall system diagram of this study. When the LED component with the frequency component is input to the RGB sensor, x-chromaticity is calculated through the filter unit, and the x-distance is estimated through the fuzzy system using this value.

**Figure 8:**
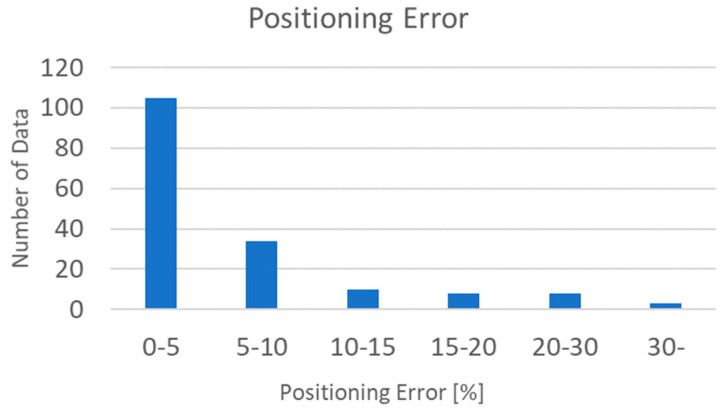
Number of data per error.

As shown in Figure 4. To estimate the lane position corresponding to the x-chromaticity, the RGB sensor was moved by 5 cm units from 5 to 60 cm along the x-axis and 5 to 80 cm along the y-axis on the floor shown in Figure 2. The measurement results are listed in Table 2. Furthermore, to analyze the lane positioning error, the percentile errors of positioning were calculated in terms of x-distance (cm). The maximum error occurred when the x-distance was 5 cm, and the mean error was about 22.8%. The minimum error occurred when the x-distance was 60 cm, with a mean error of about 1.4%. When the errors of the first lane were excluded, the mean error was smaller than 4.86% (0.5 cm), which is smaller than the errors reported in other positioning studies [1,2,3]. Figure 8 shows the error distribution of the data. The average error rate is about 4.86%, and the almost data distribution falls within 10%, especially 0–5%, and most of the large errors occurred around 5 cm of the x-distance.

## 5. Conclusions

In conclusion, the RGB stimulus values of LED lights were analyzed and a lane positioning system for autonomous vehicles was developed. The newly developed positioning system was also verified through experimental studies. LED lamps with three color temperatures of 3000 K, 4500 K, and 6000 K were used. The color temperatures of the lamps were mixed and overlapped, which changed the chromaticity. These changes in chromaticity were used, and the position was measured using the x-chromaticity value, which showed a larger change than the y-chromaticity value. To receive a signal differentiated from other disturbances, a specific frequency is applied to drive the LED, and as a result of the experiment using the fuzzy system, it was shown that the proposed positioning system could recognize the position well with an error level of less than 4.86% on average. It is expected that the precision of the lane positioning system under GPS-denied environments can be improved if the proposed lane positioning technology based on the chromaticity of LED street lamps is fused with the existing GPS, vision, and IMU techniques through continuous research. Besides lane positioning, two-dimensional plane positioning is also possible by arranging the LED street lamps proposed in this study under GPS-denied environments, such as inside a tunnel. Therefore, the proposed system is expected to be applicable when it is difficult to recognize lanes due to the presence of GPS interference points.

## Figures and Tables

**Table 1:** Measured x-chromaticity values.

x (cm)	Y	x-Chromaticity Value	Mean
0	Low	0.3591	0.3594
	Middle	0.3602	
	High	0.359	
10	Low	0.3614	0.3615
	Middle	0.3618	
	High	0.3612	
20	Low	0.3631	0.3634
	Middle	0.3638	
	High	0.3632	
30	Low	0.3652	0.3656
	Middle	0.3661	
	High	0.3656	
40	Low	0.3674	0.3679
	Middle	0.3682	
	High	0.3681	
50	Low	0.3695	0.3698
	Middle	0.3701	
	High	0.3697	
60	Low	0.3712	0.3713
	Middle	0.3717	
	High	0.3711	

**Table 2:** Experimental results of lane positioning.

x (cm)													
		5	10	15	20	25	30	35	40	45	50	55	60
**y (cm)**	**5**	4.44	9.53	14.9	18.2	23.9	27.7	33.3	37.4	43.1	48.2	53.7	58.9
	**10**	4.84	8.89	14.5	18.2	23.1	26.5	32.9	36.2	42	47.1	53.1	58.1
	**15**	3.62	8.33	13.6	18.8	25	30.4	35.1	40.8	44.4	50.3	53.1	58.9
	**20**	2.24	6.04	12.1	19.5	24.6	29.2	34.8	37.8	43.5	48.2	54.2	58.1
	**25**	4.04	7.33	14.9	22.2	26.1	31.5	34.1	37.4	41.4	47.6	55.8	58.1
	**30**	6.04	12.7	17.1	21.8	26.1	32.5	36.2	42	49.5	54.2	58.1	60
	**35**	7.81	12.1	17.6	22.2	24.6	32.5	36.6	42	47.6	52.5	56.3	60
	**40**	6.04	11.6	16.2	22.7	26.1	31	35.1	40	44.9	51.1	56.3	60
	**45**	6.44	11.6	15.8	21.2	25.7	31	35.5	40	45.3	51.9	55.8	60
	**50**	6.44	10.9	15.3	21.2	26.1	31.5	35.1	38.8	45.3	50.3	54.7	60
	**65**	5.63	10.2	15.8	21.8	25	30.4	35.1	38.8	44	49.5	55.2	60
	**70**	4.84	10.2	14.9	21.2	25	28.7	34.4	37.8	44.4	47.6	54.2	58.9
	**75**	4.04	8.89	14.9	20	25.4	28.2	33.7	38.3	44	48.2	54.2	58.9
	**80**	4.44	8.33	15.3	19.5	25	28.7	32.5	37	43.5	47.1	53.7	58.1

## Data Availability

Not applicable.
